# The Compilation of a Local History

**Published:** 1910-12-24

**Authors:** 


### The Practitioner's Relaxations.

### THE COMPILATION OF A LOCAL HISTORY.

Having spoken of the value of local history as a recreation, and of its fascination withal, it remains to indicate more intimately the nature of the authorities and the places where they must be sought. It is, of course, desirable to commence local history, as other things, at the beginning, and since such history as the ancient Britons composed was transmitted orally, we can expect to find nothing so early as their period. Yet not a little may be deduced in some localities from so tangible remains as their urns, weapons, and ornaments, and even coins which have been unearthed from time to time; while those skilled in philology may find traces of these ancient folk in the names of rivers, hills and woods, villages and towns. For the Roman period similar sources may be sought for, with, in addition, the more exact information conveyed by actual inscriptions on stone, and the remains of domestic and military buildings. Passing on to Saxon times there is an abundance of documentary evidences for local history to be found, of a period anterior even to the date of ancient Domesday, embodied in charters and grants of land to religious houses.

### The Record Office.

Kemble's 'Codex' contains a great number of transcripts of such documents, and the cartularies of the various monastic foundations will be found to include many others. Considerable numbers of originals exist in the Harleian and other collections in the British Museum, in the Bodleian, and the Public Record Office, while Dugdale's "Monasticon" contains many transcripts of these ancient charters. For the early Norman period, Domesday, of course, is a fount of invaluable information concerning names of persons and places; and facsimiles and transcripts of it have been published, of the whole as well as of particular counties. Of this and the succeeding mediæval period the cartularies of the various monastic houses, which continued to increase and multiply in the land, are even more full of information for the local historian than those of the Saxon era. The court-rolls of an infinite number of manors up and down the country, and the minister's accounts in connection therewith, also afford an inexhaustible supply of details relating to places and persons. These may be found at the British Museum, in the Harleian and Cottonian MSS., as well as in the Bodleian and Lambeth Palace Libraries and the Public Record Office. The latter, indeed, is the fountain head of material for local history. Situate in Chancery Lane, it is extremely "get-at-able" and free from irksome or troublesome regulations for access to its riches. One has merely to sign name and address in the book lying open in the entrance hall, to turn to the right and pass along the corridor to the search-room. Its walls are lined with indexes, calendars, catalogues, from those in seventeenth or eighteenth century manuscript to the latest printed calendar of patent rolls. Having searched the index or calendar of any group of documents for the earliest reference to the place or person desired, it is then merely necessary to enter on one of the forms kept at the official desk the title and number of document needed, and in due course it will be handed to the reader to be searched at his will. The later calendars of the patent rolls are of extreme value to the local historian, quite superseding the single folio printed in 1800, not that this is to be despised, if only for the fact that it may be picked up occasionally from a second-hand bookseller or at auction for a small sum of money compared with the cost of the many volumes recently issued. Besides these, the Close, Charter, Originalia, Assize, Subsidy, Hundred, and Parliament Rolls are full of information concerning persons, places, and things; as also are the Inquisitiones Post Mortem, Nonarum, and Ad Quod Damnum, and the numerous surveys of Crown Lands, manors, and monasteries, and perambulations of forests.

### Old Documents.

It would occupy too much space to describe exactly the nature of this great variety of documents, but the inquirer will find this admirably done in Sims' "Manual for the Genealogist, Topographer, and Antiquary," of which, naturally, the most recent edition is the most helpful, though even the original issue is of such great service that it should not be despised in default of the latest. In addition to all these documents, ancient wills are a valuable repository of information regarding persons and places and items of the local church history, concerning its fabric, furniture, endowments, and dedication, in not a few cases unmentioned elsewhere. As an instance, I may mention the "last will and testament" of a parishioner of a Sussex village, who, under date 1499, directs his "body to be buried in the chapel annexed to the parish church, which I edified and bilded," and who also leaves money for the singing of Masses at the "Scala Celi" in Lewes Priory. Though much has been written about this church, and more still about the Priory, the origin of the first-mentioned structure, or the very existence of the latter, was quite unknown until I discovered them in this ancient will. A Scala Cœli, or "Ladder of Heaven," it may be said, was a stairway in the neighbourhood of the high altar in church or cathedral, which the devout were wont to ascend for their oraisons; only three or four are known to have existed in England. Another ancient will, two hundred years older than the aforesaid, mentions the testator's "houses on the bridge of Lewes," and gives a number of mediæval place-names in neighbouring parishes.

To exhaust so great a variety of documents would fill the leisure of a long-lived practitioner.

